# Commentary: Short-Chain Alcohols Upregulate GILZ Gene Expression and Attenuate LPS-Induced Septic Immune Response

**DOI:** 10.3389/fimmu.2020.00728

**Published:** 2020-04-21

**Authors:** László Pál, Orsolya Bujdosó, Martin McKee, János Sándor, Sándor Szűcs

**Affiliations:** ^1^Department of Preventive Medicine, Faculty of Public Health, University of Debrecen, Debrecen, Hungary; ^2^European Centre on Health of Societies in Transition, London School of Hygiene and Tropical Medicine, London, United Kingdom

**Keywords:** short chain alcohols, aliphatic alcohols, granulocytes, monocytes, phagocytosis, immunosuppression, immunotoxicity, alcohol abuse

We read with great interest the paper by Ng et al. on the effects of short chain alcohols on glucocorticoid-induced leucine zipper (GILZ) gene expression and lipopolysaccharide-induced (LPS) cytokine production by Human Mono-Mac-6 cells and LPS-induced septic immune response in C57BL/6 mice (1). The authors concluded that short chain alcohols, such as ethanol, propanol and isopropanol (2-propanol), upregulate GILZ gene expression, and suppress host inflammatory response to LPS in a dose dependent manner (1). These findings are potentially important because these processes play an important role in host defense mechanisms against pathogenic microorganisms (2–4). Based on their findings the authors suggested that short chain alcohols can be used to alleviate LPS-induced septic shock (1). However, we believe that the results published by Ng et al. are not only of clinical but also of public health relevance. Short chain alcohols, also known aliphatic alcohols (AAs), including methanol and higher alcohols containing more than two carbon atoms such as 1- and 2-propanol, 1- and 2-butanol, isobutanol, and isoamyl alcohol are found in many legally and illegally produced alcoholic beverages, including beer, wine, and spirits (5–12). Very large portion of the population is potentially exposed to these substances, with the latest available data from the World Health Organization, from 2016, reporting that 2.34 billion people worldwide consume alcoholic beverages, with a global annual average adult per capita alcohol consumption from legal and illegal sources was 4.7 and 1.6 l, respectively (13). Therefore, toxicologists and public health researchers have expressed concern about potential health risks associated with consumption of alcoholic beverages containing AAs (5–10). Although AAs are present in all types of alcoholic beverages, their concentration is typically higher in distilled spirits than in beer and wine (6, 12).

The adverse effects of ethanol on the liver, gastrointestinal, nervous, and cardiovascular systems are well-known but acute and chronic alcohol consumption have also been found to cause immunotoxicity (14–21). Consequently, heavy alcohol use has been associated with increased susceptibility to bacterial and viral infections and higher morbidity and mortality from a number of infectious diseases including tuberculosis, pneumonia, human immunodeficiency virus-1, and hepatitis C infections (16, 19, 20). While the health effects of ethanol consumption alone are an important public health concern, less attention has been paid to the possible immunotoxic effects of AAs found in alcoholic beverages. We have previously shown that AAs can decrease superoxide-anion production by human granulocytes and inhibit phagocytosis by human granulocytes and monocytes in a concentration dependent manner and, when combined with ethanol, they caused a further decrease in phagocytic activity (22–24). We have found similar effects when granulocytes and monocytes were exposed to metabolites of AAs including formaldehyde, 1-propanal, acetone, 1-butanal, and 2-butanone (25). We have also calculated that blood concentrations of AAs and their metabolites could reach levels sufficient to decrease phagocytosis by granulocytes and monocytes in a clinically meaningful way in heavy users of distilled spirits (24, 25). Thus, AAs and their metabolites may contribute to increased susceptibility to infectious diseases (22–25). Our population-based risk assessment demonstrated that, in addition to ethanol, exposure to methanol, 1-propanol, 1-butanol, and isoamyl alcohol from distilled spirits posed a health risk for heavy alcohol users (26). In light of these and previous findings on immunotoxic effects of AAs, the results obtained by Ng et al. deserves wider attention.

In conclusion, we encourage the authors to extend their research to provide a better understanding of the role of AAs separately and in combination in molecular and cellular mechanisms responsible for short chain alcohol-induced immunotoxicity.

## Author Contributions

LP, OB, MM, JS, and SS have made a substantial contribution to the work and approved it for publication.

## Conflict of Interest

The authors declare that the research was conducted in the absence of any commercial or financial relationships that could be construed as a potential conflict of interest.
